# Fimasartan reduces clinic and home pulse pressure in elderly hypertensive patients: A K-MetS study

**DOI:** 10.1371/journal.pone.0214293

**Published:** 2019-04-09

**Authors:** Eun Joo Cho, Ki Chul Sung, Seok Min Kang, Mi-Seung Shin, Seung Jae Joo, Jeong Bae Park

**Affiliations:** 1 Division of Cardiology, St. Paul’s Hospital, Catholic University of Korea, Seoul, Korea; 2 Division of Cardiology, Kangbuk Samsung Hospital, Sungkyunkwan University, Seoul, Korea; 3 Division of Cardiology, Severance Cardiovascular Hospital, Yonsei University, Seoul, Korea; 4 Division of Cardiology, Gachon University Gil Medical Center, Incheon, Korea; 5 Division of Cardiology, Jeju National University Hospital, Jeju, Korea; 6 JB lab and clinic, Seoul, Korea; 7 Sungkyunkwan University School of Medicine, Seoul, Korea; Shanghai Institute of Hypertension, CHINA

## Abstract

**Background:**

Angiotensin II receptor blockers (ARBs) are recommended for treating patients with hypertension. However, comparative safety and efficacy of ARB use in elderly patients have not been well established. This study was designed to determine the efficacy of fimasartan, an ARB, in hypertensive elderly patients by measuring clinic and home blood pressures (BPs) and evaluating safety compared to nonelderly patients.

**Method:**

In the K-MetS study, a nationwide prospective observational study of hypertensive patients on fimasartan, elderly patients (60 years and older) who were treated for 1 year with fimasartan were recruited. BP was evaluated in clinic and at home.

**Results:**

Of the 6 399 enrolled patients, 2 363 were elderly (46.9% males, mean age 67.3 ± 5.7 years). Fimasartan reduced systolic and diastolic BP (SBP and DBP) in clinic from 144.1 ± 17.3 to 127.7 ± 12.9 mmHg and from 85.1 ± 10.4 to 76.8 ± 8.4 mmHg, respectively, (all p<0.0001) in 1 year. Similar results were found in home BPs. These BP changes were consistent with those in nonelderly patients. However, pulse pressure, a better predictor of cardiovascular events in the elderly, decreased more in elderly than in nonelderly patients by -8.2 ± 0.3 in elderly and -7.0 ± 0.2 mmHg (p<0.0001), respectively, after adjustment for confounding factors. Adverse events were reported in 1.6% of elderly hypertensives, independent of dose, which was consistent with results in nonelderly patients.

**Conclusions:**

Fimasartan resulted in better pulse pressure reduction with similar BP reduction efficacy and safety in hypertensive elderly patients compared with nonelderly patients.

## Introduction

The prevalence of hypertension increases with age, and the worldwide prevalence of hypertension in people older than 70 years is approximately 70%. [1] Hypertension is an important public health issue with substantial societal burden because it is a leading risk factor for cardiovascular disease (CVD), which is a primary cause of total mortality. [2] A meta-analysis of large cohort studies conducted worldwide also found linear relationships between cardiovascular risk and blood pressures (BPs) irrespective of age. [3]

Treatment of hypertension reduces CVD and total mortality in elderly patients [4], although uncertainty about the optimal systolic BP (SBP) remains. In 2014, the 8^th^ Joint National Committee (JNC8) recommended a target SBP less than 150 mmHg and diastolic BP (DBP) less than 90 mmHg for patients older than 60. [5] However, to reduce cardiovascular risks, BP should be kept as low as tolerated, as evidenced by numerous clinical trials and epidemiological data as well as the latest guidelines. [6 – 8]

In older hypertensive patients, pulse pressure (PP), not mean pressure, is the major determinant of cardiovascular risk [9] and heart failure. [10] A more targeted approach to lowering PP would come from an increase in arterial compliance; studies have suggested that angiotensin-converting enzyme inhibitors have a direct effect on arterial walls. [11–15]

Fimasartan is the 9^th^ angiotensin II receptor blocker (ARB) and has been proven safe and effective in reducing BP in the Safe-KanArb and K-MetS studies. [16, 17] This study was designed to determine the safety and efficacy of fimasartan and evaluate extent of BP control level in the clinical setting using elderly patients with hypertension enrolled from primary care clinics and tertiary care hospitals.

## Methods

Elderly hypertensive patients enrolled in this study were selected from participants in the K-MetS study, a nationwide prospective, multicenter, single-arm, observational study evaluating patients from primary care clinics to tertiary care hospitals on antihypertensive treatment including fimasartan. According to population-based surveys, available data [18–21], and JNC8 guidelines, we defined elderly as older than 60 years. This study was registered at https://cris.nih.go.kr/cris/en, CRIS: KCT0000529.

The study design, socioeconomic and demographic characteristics of the participants, and details of the project have been described in our previous article. [22] This study was approved by the Institutional Review Board Committee at the Cheil General Hospital, Dankook University College of Medicine, on behalf of 582 primary care clinics. Another 10 university hospitals in Korea approved this study through their own institutional review board committees. Written informed consent was obtained from all study subjects.

### Study population

A total of 6 399 patients, including elderly patients, who were treated for 1 year with fimasartan (30–120 mg daily) were recruited from 582 primary care clinics and 11 university hospitals between October 17, 2011 and October 31, 2012. The data were systematically collected using electronic case report forms. Patients were required to 1) have hypertension, be at least 20 years of age, and intend to use fimasartan, 2) agree to participate in the study and sign the informed consent form, and 3) be in the fasting state at each visit. Patients who were treated with fimasartan at baseline were excluded. Enrolled patients completed 3 months and 1 year follow-up visits and scheduled BP measurements. Of these, 2 363 (36.9%) were elderly patients (S1 Fig).

### Measurement of BP

The upper arm cuff devices based on the oscillometric principle were used to measure BP. The Omron HEM-7220 was used to measure clinic BP, and the Omron HEM-7200 was used to measure home BP (both Omron, Tokyo, Japan). [23] Clinic BP measurements were performed under standardized conditions (in the same arm by the same physician or nurse). Clinic BP was measured at least twice within a 2-minute interval on the same arm at each visit after a 5-minute seated rest. The average value of the two measurements was used for analysis. [24] The study participants or their family members were educated about self-BP measurement at home and provided with the follow instructions: measure the BP twice a day (in the morning and evening) and record an average of three consecutive BP readings at 2-minute intervals on each occasion from the same arm for seven consecutive days. Morning BP was measured within 1 hour of awakening, after urination, in the sitting position, after resting for 5 minutes, and before taking medications or eating. In the evening, BP was measured before going to bed, after resting for 5 minutes, and in the sitting position. Data from the first day was excluded, and an average of BP measurements from the remaining 6 days was used for the analysis. Assessments, including data from a health questionnaire and BP measurements, were obtained at the start of the study and at 3 months and 1 year after initiating treatment with fimasartan.

### Data analysis

Characteristics of study subjects were compared using the χ^2^ test for dichotomous variables or the independent t-test for continuous variables. Differences between measured variables (e.g., BP) were examined using the repeated measure analysis of variance at baseline and at 3 months and 1 year of follow up. Relative risk estimation of achievement and adverse events between elderly patients and nonelderly patients was calculated using 2x2 tables. The adjusted means of decrease in systolic and diastolic BP were compared between the groups after adjusting for sex, body mass index, diabetes mellitus, alcohol, and smoking. All continuous values were expressed as the mean ± the standard deviation, and categorical values were presented as frequencies and percentages (%). A p value of less than 0.05 was considered statistically significant. All analyses were performed using SAS 9.4 (SAS Institute, Cary, NC, USA).

## Results

### Subject characteristics

Of the elderly patients, 800 (34%) had newly diagnosed hypertension started on fimasartan, 940 patients (40%) were switched from other antihypertensive drugs to fimasartan, and 614 (26%) were started on fimasartan in addition to their prior antihypertensive medication regimen. Table 1 shows baseline characteristics and laboratory data of subjects. Among the total elderly patients initially recruited, 2 363 (mean age 67.3 ± 5.7 years old, 1 109 were male [46.9%]) were successfully followed for 1 year. Overall, fimasartan reduced clinic SBP from 144.1 ± 17.3 to 127.7 ±1 2.9 mmHg and clinic DBP from 85.1 ± 10.4 to 76.8 ± 8.4 mmHg (all p<0.0001) (Table 2). The heart rate was reduced from 72.7 ± 10.3 to 71.5 ± 9.2 beats/min at 1 year of fimasartan treatment (p<0.0001). This efficacy of fimasartan in elderly patients was consistent regardless of patient’s sex, underlying medical condition, or comorbidities.

**Table 1:** Baseline characteristic (Age ≥ 60yr vs. Age < 60yr).

	Total (n = 6399)	Age ≥ 60yr (n = 2363)	Age < 60yr (n = 4036)	p-value
Mean age, years	56.3 ± 10.6	67.3 ± 5.7	49.8 ± 6.7	< .0001
Sex, female (%)				< .0001
Male, (%)	3329(51.6)	1109(46.9)	2190(54.3)	
Female, (%)	3100(48.5)	1254(53.7)	1846(45.7)	
Body weight, kg	67.2 ± 11.4	63.9 ± 9.6	69.2 ± 12.0	< .0001
Height, cm	162.9 ± 8.6	160.3 ± 8.4	164.3 ± 8.4	< .0001
Body mass index, kg/m^2^	25.3 ± 3.2	24.8 ± 3.0	25.5 ± 3.3	< .0001
Smoking, (%)	1138(17.8)	252(10.7)	886(22.0)	< .0001
Alcohol use, (%)	2864(44.8)	775(32.8)	2089(51.8)	< .0001
**Blood Parameters**				
**Baseline**				
Na, mg/dL	140.5 ± 4.2	140.9 ± 4.1	140.3 ± 4.2	< .0001
K, mg/dL	4.9 ± 0.8	4.9 ± 0.8	4.9 ± 0.8	0.3787
Uric acid, mg/dL	5.2 ± 1.5	5.1 ± 1.5	5.2 ± 1.5	0.0035
eGFR, mg/dL	82.1 ± 17.5	76.0 ± 17.1	85.6 ± 16.7	< .0001
Creatinine, mg/dL	0.89 ± 0.37	0.92 ± 0.38	0.87 ± 0.35	< .0001
**3 months**				
Na, mg/dL	140.0 ± 4.0	140.4 ± 4.0	139.7 ± 3.9	< .0001
K, mg/dL	4.9 ± 0.8	4.9 ± 0.8	4.9 ± 0.8	0.8995
Uric acid, mg/dL	5.4 ± 1.5	5.4 ± 1.5	5.4 ± 1.5	0.6472
eGFR, mg/dL	80.0 ± 17.3	73.6 ± 16.7	83.7 ± 16.5	< .0001
Creatinine, mg/dL	0.91 ± 0.41	0.95 ± 0.42	0.89 ± 0.40	< .0001
**1 year**				
Na, mg/dL	140.7 ± 4.0	141.0 ± 3.9	140.6 ± 4.0	0.0006
K, mg/dL	4.8 ± 0.7	4.8 ± 0.6	4.8 ± 0.7	0.1506
Uric acid, mg/dL	5.3 ± 1.5	5.3 ± 1.5	5.3 ± 1.5	0.6755
eGFR, mg/dL*	81.2 ± 19.0	75.0 ± 18.8	84.9 ± 18.2	< .0001
Creatinine, mg/dL	0.91 ± 0.43	0.94 ± 0.41	0.89 ± 0.44	0.0001
**Medical condition (%)**				
Diabetes mellitus	1076(16.8)	527(22.3)	549(13.6)	< .0001
Coronary heart disease	473(7.4)	244(10.3)	229(5.7)	< .0001
Cerebrovascular disease	60(0.9)	45(1.9)	15(0.4)	< .0001
**Concomitant medication (%)**				
ACE inhibitor	113(1.8)	44(1.9)	69(1.7)	0.6550
Beta blocker	480(7.5)	197(8.3)	283(7.0)	0.0522
Calcium channel blocker	1602(25.0)	684(29.0)	918(22.8)	< .0001
Diuretics	273(4.3)	123(5.2)	150(3.7)	0.0045
Alpha blocker	25(0.4)	11(0.5)	14(0.4)	0.4629
Other antihypertensive drugs	79(1.2)	32(1.4)	47(1.2)	0.5072
Antiplatelet agent	1006(15.7)	555(23.5)	451(11.2)	< .0001
Oral hypoglycemic agent	887(13.9)	435(18.4)	452(11.2)	< .0001
Antidyslipidemic agent	1340(20.9)	619(26.2)	721(17.9)	< .0001
**Indication (%)**				< .0001
Naïve	2535 (39.7)	800 (34.0)	1735 (43.1)	
Switch	2483 (38.9)	940 (39.9)	1543 (38.3)	
Add-on	1361 (21.3)	614 (26.1)	747 (18.6)	

Na, sodium; K, potassium; eGFR, estimated glomerular filtration rate; ACE, angiotensin converting enzyme; c-, clinic; h-, home

*eGFR was calculated using IDMS-traceable MDRD study equation; eGFR (mL/min/1.73 m^2^) = 175 × (S_cr_)^-1.154^ × (Age)^-0.203^ × (0.742 if female) × (1.212 if African American) [25]

**Table 2:** Change of blood pressure at clinic and home during 1 year follow-up.

	Age ≥ 60yr (n = 2363)				Age < 60yr (n = 4036)				
	Baseline	3 months	1 year	p-value	Baseline	3 months	1 year	p-value	Age≥60yr vs. Age<60yr
**Clinic blood pressure**									
SBP, mmHg	144.1 ± 17.3	128.2 ± 13.5*	127.7 ± 12.9^†^^‡^	< .0001	144.1 ± 16.9	127.4 ± 12.3*	126.7 ± 11.7^†^^‡^	< .0001	0.0869
DBP, mmHg	85.1 ± 10.4	77.3 ± 8.8*	76.8 ± 8.4^†^^‡^	< .0001	90.4 ± 11.4	80.8 ± 9.0*	79.9 ± 8.4^†^^‡^	< .0001	< .0001
Pulse rate, bpm	72.7 ± 10.3	71.3 ± 9.1*	71.5 ± 9.2^†^	< .0001	74.5 ± 10.1	73.0 ± 9.5*	72.8 ± 9.4^†^	< .0001	0.1193
Pulse pressure, mmHg	59.0 ± 13.1	50.8 ± 10.8*	50.9 ± 10.6^†^	< .0001	53.7 ± 11.6	46.6 ± 8.9*	46.8 ± 8.8^†^	< .0001	0.0027
**Isolated systolic hypertension**	n = 627				n = 605				
SBP, mmHg	151.3 ± 10.1	130.5 ± 13.7*	129.6 ± 13.3^†^	< .0001	148.8 ± 8.0	128.8 ± 11.3*	127.7 ± 11.3^†^	< .0001	0.7550
DBP, mmHg	82.1 ± 5.6	75.8 ± 8.5*	75.1 ± 8.2^†^	< .0001	83.8 ± 4.8	78.2 ± 7.8*	77.7 ± 7.8^†^	< .0001	0.0854
Pulse pressure, mmHg	69.3 ± 10.9	54.7 ± 11.8*	54.5 ± 11.6^†^	< .0001	65.1 ± 9.1	50.6 ± 9.4*	49.9 ± 9.2^†^	< .0001	0.5468
**Home blood pressure**									
**SBP, mmHg**									
6day-average (all)	139.5 ± 19.4	128.0 ± 15.6*	126.1 ± 12.2^†^	< .0001	136.8 ± 19.2	124.7 ± 14.5*	123.0 ± 10.2^†^^‡^	< .0001	0.8885
6day-average (day)	140.4 ± 19.5	128.5 ± 16.5*	126.3 ± 12.9^†^^‡^	< .0001	137.2 ± 19.2	125.4 ± 14.9*	123.0 ± 10.7^†^^‡^	< .0001	0.9729
6day-average (night)	138.7 ± 20.5	127.5 ± 15.7*	126.0 ± 12.8^†^	< .0001	136.4 ± 20.2	124.0 ± 15.0*	123.1 ± 10.8^†^	< .0001	0.5968
**DBP, mmHg**									
6day-average (all)	78.5 ± 11.9	72.5 ± 9.2*	71.8 ± 8.6^†^	< .0001	84.4 ± 12.2	77.1 ± 10.4*	76.2 ± 7.7^†^^‡^	< .0001	0.0731
6day-average (day)	79.5 ± 12.0	73.2 ± 9.5*	72.3 ± 8.9^†^	< .0001	85.3 ± 12.4	78.1 ± 10.6*	76.5 ± 8.2^†^^‡^	< .0001	0.0902
6day-average (night)	77.6 ± 12.4	71.8 ± 9.5*	71.4 ± 9.0^†^	< .0001	83.5 ± 12.7	76.1 ± 10.8*	75.6 ± 8.0^†^	< .0001	0.0543
**Pulse rate, bpm**									
6day-average (all)	71.0 ± 11.1	70.7 ± 9.8	70.1 ± 8.9	0.3105	73.5 ± 11.6	72.2 ± 10.2*	71.5 ± 8.2^†^	< .0001	0.3040
6day-average (day)	69.8 ± 11.2	67.0 ± 10.5	69.2 ± 9.4	0.5801	72.9 ± 11.8	71.6 ± 10.4*	71.0 ± 8.6^†^	0.0002	0.1083
6day-average (night)	72.3 ± 11.5	71.4 ± 10.0*	71.0 ± 8.9^‡^	0.0700	74.1 ± 11.9	72.88 ± 10.53*	72.13 ± 8.48^†^	0.0003	0.4233
**Pulse pressure, mmHg**	61.7 ± 13.7	55.6 ± 11.9*	53.5 ± 10.8^†^^‡^	< .0001	54.1 ± 11.4	49.1 ± 9.7*	47.1 ± 7.5^†^^‡^	< .0001	0.0967

SBP, systolic blood pressure; DBP, diastolic blood pressure

*: baseline vs. 3 months,

^†^: baseline vs. 1years,

^‡^: 3 months vs. 1year

### Clinic BP monitoring

**Fig 1:**
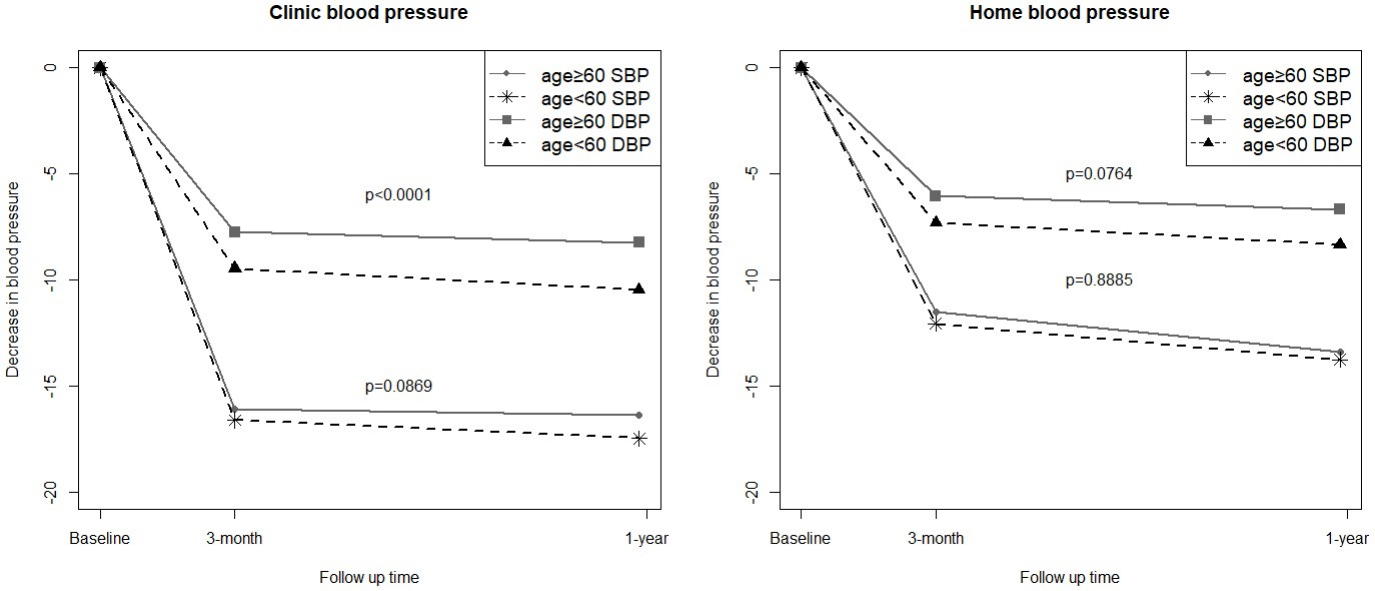
BP reduction over time during the 1 year of fimasartan treatment in elderly vs. nonelderly patients. Elderly patients exhibited a lesser and more gradual decline in clinic DBP than nonelderly patients during the 1 year of fimasartan treatment (left panel). Elderly patients also exhibited a lesser and more gradual decline in home DBP than nonelderly patients during the 1 year of fimasartan treatment, also the difference was not statistically significant (right panel). P value was obtained by repeated measures analysis of variance. Abbreviations: BP, blood pressure; DBP, diastolic BP; SBP, systolic BP.

Both clinic and home BP were significantly reduced at 1 year of fimasartan treatment (Fig 1). Elderly patients exhibited a lesser and more gradual decrease in DBP during 1 year of fimasartan treatment than nonelderly patients (Fig 1, left panel). The changes of blood pressure at clinic and home was generally more prominent in low doses of 30 and 60mg than higher dose of 120mg (S2 Table).

### Home BP monitoring

Table 2 shows the results of 6-day averaged day and night home BP. The 6-day averaged day and night SBP, DBP, and heart rate were also significantly reduced in elderly patients at 1 year of fimasartan treatment. The BP reduction in elderly patients was consistent regardless of fimasartan indications. As with SBP, elderly patients exhibited a lesser and more gradual reduction in DBP during 1 year of treatment with fimasartan than nonelderly patients (Fig 1, right panel).

### Pulse pressure changes during 1 year of treatment

**Fig 2:**
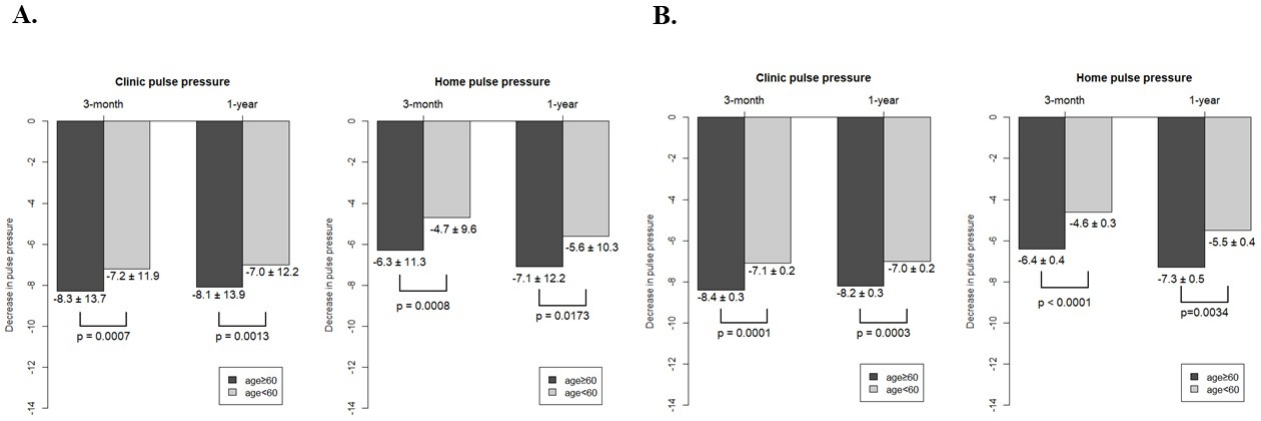
Bar graphs depicting comparison of pulse pressure reduction between elderly vs nonelderly patients. (A) Both clinic (left panel) and home (right panel) pulse pressure reductions were significantly greater in elderly patients at 3 months and 1 year following initiation of fimasartan therapy. (B) Even after adjusting for sex, body mass index, diabetes mellitus, alcohol and smoking, clinic (left panel) and home (right panel) pulse pressure decreased significantly greater in elderly patients for 3 months and 1 year follow-up. *Adjust for sex, body mass index, diabetes mellitus, alcohol and smoking.

Pulse pressure at baseline and at 3 months and 1 year of treatment are shown in Table 2. Both elderly and nonelderly patients exhibited a significant reduction in pulse pressure at clinic and home. The clinic pulse pressure was higher in elderly patients compared with nonelderly patients (p = 0.0027). The magnitude of clinic and home pulse pressure reduction between baseline and 3 months of treatment and between baseline and 1 year of treatment was more prominent in elderly versus nonelderly patients, when adjusted for sex, body mass index, diabetes mellitus, alcohol, and smoking (Fig 2). PP reduction in naïve patients was statistically significantly higher in elderly patient than in nonelderly patients in clinic and home between baseline and 3 months and between baseline and 1 year. This effect found persistently through 1 year (S3 Table). And this effect was found in clinic BPs in switch patients but not in add-on patients (Table 3). In isolated hypertension, pulse pressure reduction at clinic and home was 14.5 ± 13.5mmHg and 8.4 ± 12.6 mmHg for 3 months and 14.8 ± 13.3 mmHg and 9.8 ± 12.7 mmHg for 1 year. Home pulse pressure reduction was more prominent in elderly than in nonelderly.

**Table 3:** Decrease in pulse pressure for 1 year after adjustment by indication group.

Indication		Mean ± SD			Adjusted* mean ± SD		
		Age ≥ 60yr	Age < 60yr	p-value	Age ≥ 60yr	Age < 60yr	p-value
Naïve	Δ(Baseline—3 months)	n = 800	n = 1735				
	Clinic pulse pressure	-13.2 ± 13.3	-10.6 ± 12.1	< .0001	-13.1 ± 0.5	-10.6 ± 0.3	< .0001
	Home pulse pressure	-11.2 ± 11.0	-6.9 ± 9.2	< .0001	-11.3 ± 0.6	-6.9 ± 0.4	< .0001
	Δ(Baseline—1 year)						
	Clinic pulse pressure	-13.8 ± 13.3	-10.6 ± 12.4	< .0001	-13.7 ± 0.5	-10.6 ± 0.3	< .0001
	Home pulse pressure	-11.7 ± 11.4	-7.6 ± 9.0	< .0001	-11.6 ± 0.8	-7.6 ± 0.5	< .0001
Switch	Δ(Baseline—3 months)	n = 940	n = 1543				
	Clinic pulse pressure	-5.0 ± 12.8	-3.9 ± 10.6	0.0254	-5.1 ± 0.4	-3.9 ± 0.3	0.0196
	Home pulse pressure	-3.5 ± 9.0	-3.1 ± 9.2	0.4639	-3.5 ± 0.5	-3.1 ± 0.4	0.5883
	Δ(Baseline—1 year)						
	Clinic pulse pressure	-4.5 ± 13.0	-3.5 ± 10.9	0.0489	-4.6 ± 0.4	-3.5 ± 0.3	0.0378
	Home pulse pressure	-4.0 ± 10.0	-3.8 ± 10.2	0.8479	-4.1 ± 0.7	-3.7 ± 0.5	0.6676
Add-on	Δ(Baseline—3 months)	n = 614	n = 747				
	Clinic pulse pressure	-7.2 ± 13.8	-6.0 ± 11.7	0.0854	-7.1 ± 0.5	-6.0 ± 0.5	0.1294
	Home pulse pressure	-5.3 ± 12.6	-4.0 ± 10.4	0.2084	-5.5 ± 0.7	-3.8 ± 0.7	0.0995
	Δ(Baseline—1 year)						
	Clinic pulse pressure	-6.3 ± 13.7	-6.0 ± 11.9	0.6255	-6.1 ± 0.5	-6.1 ± 0.5	0.9730
	Home pulse pressure	-7.1 ± 13.7	-5.9 ± 11.7	0.3186	-7.4 ± 0.9	-5.7 ± 0.8	0.1797

*Adjust for sex, body mass index, diabetes mellitus, alcohol and smoking.

### Target BP achievement rate

Using a target of SBP<150 mmHg and DBP<90 mmHg for clinic BP in age ≥ 60 (JNC 8 guideline) [5], 2 117 elderly subjects (89.6%, p<0.0001 vs. nonelderly patients) achieved the goal. Using the latest guidelines, the 2018 European Society of Cardiology (ESC) and European Society of Hypertension (ESH) guideline (SBP<140 mmHg and DBP < 80 mmHg in age ≥ 65) [7], 778 elderly patients (53.4%, p<0.0001 vs. nonelderly patients) and the 2017 American College of Cardiology (ACC) and American Heart Association (AHA) guideline (SBP<130 mmHg and DBP < 80 mmHg in age ≥ 65) [8], 590 elderly patients (40.5%, p = 0.6745 vs. nonelderly patients) achieved their goals. According to 2014 JNC 8 and 2018 ESC / ESH guidelines, the goal achievement rate was statistical significantly higher in elderly patients than in nonelderly patients. However, the achievement rate according to the 2017 ACC/AHA guideline was not different between the elderly and nonelderly patients (S4 Table).

### Adverse events

Of the elderly patients, 37 (1.6%) experienced adverse events. Adverse events were not dose-dependent. There was no difference in the incidence of adverse events between the elderly and the nonelderly patients (Table 4). The most frequent adverse event was dizziness (7 subjects, 0.3%), and none of these events led to drug discontinuation in elderly patients.

**Table 4:** Adverse event.

Adverse event	Total	Age ≥ 60yr	Age < 60yr
**Central and peripheral nervous system disorders**			
No event	6365 (99.5)	2354 (99.6)	4011 (99.4)
Event	34 (0.5)	9 (0.4)	25 (0.6)
**Relative risk = 0.61 (0.25–1.36), p = 0.2846**			
**Musculoskeletal system disorders**			
No event	6382 (99.7)	2358 (99.8)	4024 (99.7)
Event	17 (0.3)	5 (0.2)	12 (0.3)
**Relative risk = 0.71 (0.20–2.17), p = 0.6209**			
**Digestive system disorders**			
No event	6381 (99.7)	2356 (99.7)	4025 (99.7)
Event	18 (0.3)	7 (0.3)	11 (0.3)
**Relative risk = 1.09 (0.36–3.08), p = 1.0000**			
**Respiratory disorders**			
No event	6381 (99.7)	2357 (99.8)	4024 (99.7)
Event	18 (0.3)	6 (0.3)	12 (0.3)
**Relative risk = 0.85 (0.26–2.46), p = 0.8125**			
**Others**			
No event	6378 (99.7)	2353 (99.6)	4025 (99.7)
Event	21 (0.3)	10 (0.4)	11 (0.3)
**Relative risk = 1.55 (0.59–4.04), p = 0.3661**			
**Total**			
No event	6291 (98.3)	2326 (98.4)	3965 (98.2)
Event	108 (1.7)	37 (1.6)	71 (1.8)
**Relative risk = 0.89 (0.58–1.34), p = 0.6157**			

## Discussion

This research shows that fimasartan treatment was as safe and effective at BP reduction in elderly patients as in nonelderly patients, even beyond the target level, as assessed by clinic BP and home BP measurements. Furthermore, the average level of BP control in elderly hypertensives started on fimasartan was not significantly different from that in nonelderly patients started on the same treatment. However, pulse pressure reduction in elderly patients was greater during the treatment period. Because the majority of subjects were enrolled from primary care clinics, these results reflect the actual status of treatment in elderly hypertensive patients.

A Cochrane review that included 15 studies with 24,000 subjects found that treatment of hypertension improves rates of cardiovascular and cerebrovascular morbidity and mortality in elderly patients. [26] However, the optimal SBP goal in elderly patients is not certain. Treatment of hypertension in elderly patients is also more complicated than in nonelderly patients. As arterial BP increases with age, the average DBP increases from early adulthood until the end of the fifth decade. However, mean DBP decreases from the sixth decade on, and pulse pressure becomes wider with the advancement of age. [27] Also, vasculature in elderly patients is stiff; therefore, BP fluctuation might be exaggerated during the circadian rhythm in elderly patients on antihypertensive medications. [28] Pulse pressure is associated with a higher risk of CVD in elderly persons. Isolated systolic hypertension is common among the elderly and is accompanied by wide pulse pressure. Treatment of isolated systolic hypertension may further increase pulse pressure if the DBP is lowered to a greater extent than the SBP. [29] For these reasons, antihypertensive-drug-induced orthostatic hypotension is a frequent adverse event that leads to poor drug compliance and discontinuation. Research has demonstrated that the associations between orthostatic hypotension and coronary heart disease and stroke were significant for both the middle-aged and elderly participants. [30] The J-curve phenomenon associated with excessive DBP reduction in elderly patients has been reported in the Practitioner’s Trial on the efficacy of antihypertensive treatment in the elderly. [31] Therefore, antihypertensive medication that reduces SBP while maintaining DBP, thereby reducing pulse pressure, is considered ideal for elderly patients with hypertension.

From the evidence that a goal SBP of less than 140 mmHg in elderly patients provides no additional benefit compared with a higher goal of 140 to 160 mmHg or 140 to 149 mmHg [32, 33], the 8^th^ Joint National Committee (JNC8) recommended a target SBP goal of less than 150 mmHg and DBP goal of less than 90 mmHg for the treatment of hypertension in elderly patients. [5] However, numerous clinical trials and epidemiological data, even from elderly patients, have demonstrated that keeping BP as low as possible is beneficial to reducing cardiovascular risks. [6] SBP rather than DBP is more relevant for determining cardiovascular risk in patients with hypertension, particularly in those older than 50 years of age. [34–36] Findings from the Systolic Blood Pressure Intervention Trial (SPRINT) showed that reduction of SBP to less than 120 mmHg, as measured by automated office BP (AOBP), is associated with a lower incidence of CVD, cardiovascular-related mortality, and even overall mortality as compared with a reduction to less than 140 mmHg in patients greater than 50 with a high CVD risk. [37] Interestingly, the authors of this trial exerted considerable effort to minimize the white coat effect for in clinic measurements. Although there are significant advantages in cardiovascular outcomes from BP reduction beyond the value recommended in current guidelines for elderly patients, serious adverse events, such as hypotension, acute renal failure, and diuretic-induced electrolyte imbalance, were also reported. [37] Additionally, the Heart Outcomes Prevention Evaluation (HOPE)-3 trial demonstrated no reduction in the rate of cardiovascular events with antihypertensive therapy in patients with intermediate CVD risk and BP less than 140/90 mmHg at baseline. [38] In the post-SPRINT and -HOPE-3 era, new guidelines recommend a target BP of 130/80 mmHg for hypertensive patients, including elderly patients, with various comorbidities. Also, new guidelines acknowledge the 10 mmHg gap between home and office SBP and suggest that AOBP measurement be used to achieve strict BP control.

In this study results, we found that fimasartan reduced both SBP and DBP to the same extent in elderly patients as in nonelderly patients and even beyond the target BP recommended by JNC 8 without serious adverse events. The SBP gaps between clinic SBP and home SBP at baseline were about 5–8 mmHg in both age groups. The further reduction of BP was gradual and lasted at least throughout the 1 year of fimasartan treatment. However, unlike SBP reduction, fimasartan reduced DBP in elderly patients to a certain level without further significant reduction beyond the safety margin during the 1 year follow up period. Therefore, the pulse pressure reduction in elderly patients was significantly greater than that in nonelderly patients. Pulse pressure reduction were continuously decreased to 1 year at home but not in clinic. This difference may come from the difference between baseline mean home and clinic BP and from different characteristics of two BPs, both of which can’t be explained the reasons from our data (S5 Table). [39] Unlike the clinic heart rate, which was significantly reduced with fimasartan, the home-monitored heart rate was not significantly reduced during the follow up period. These effects of optimal reduction in SBP, DBP, and heart rate encourages compliance in elderly patients. Also, reduction of pulse pressure favorably affects CVD risks. The mechanism underlying fimasartan’s ability to reduce DBP and heart rate but not beyond the safety margin needs to be investigated. Orally administered fimasartan is rapidly absorbed, and enterohepatic recycling likely contributes to its long half-life. [40] Advantageous pharmacokinetics and metabolic profiling might contribute to the sustained continuing pharmacologic effect of fimasartan on stiffer vasculature, thereby preventing BP from fluctuating and possibly exerting a greater effect on SBP in elderly patients. Our study results also revealed an approximately 7–8 mmHg SBP gap at baseline between clinic and home BP, regardless of age and prescription type. Those SBP gaps disappeared by 1 year of fimasartan therapy.

Our study had several limitations. This study was observational with great heterogeneity of subjects: varying comorbidities and dissimilar pre-existing medication regimens. Very elderly patients were not included on this study. Study bias cannot be excluded because there were no controls in this analysis. However, the large number of patients enrolled in this study may overcome this limitation. Moreover, an observational study may have more relevance to real clinical practice.

In conclusion, fimasartan was as safe and effective in controlling BP in elderly patients as in nonelderly patients. Elderly patients exhibited a significantly greater reduction in pulse pressure compared with nonelderly patients. With the development of new antihypertensive agents, differentiation in hypertension treatment strategy based on age may not be appropriate. And in this study, other drug effects on the base of fimasartan were not analyzed, but drug percentage between baseline and 1 year was similar (Table 1 and S1 Table). Therefore, this suggests that the main effect of BP changes comes from fimasartan, however, which needs to find in another analysis.

## Supporting information

S1 Fig Flow chart of subject selection. Abbreviation: HBP, high blood pressure.
(TIF)

S2 Fig Pulse pressure reduction over time during the 1 year of fimasartan treatment in elderly vs. nonelderly patients. The change in clinic (left panel) and home (right panel) pulse pressure at baseline, 3 months, and 1 year are depicted above. The reduction in clinic pulse pressure between baseline and 3 months and between baseline and 1 year were greater in elderly versus nonelderly patients, when adjusted for sex, body mass index, diabetes mellitus, alcohol, and smoking. P value was obtained by repeated measures analysis of variance. Abbreviation: PP, pulse pressure.
(TIF)

S1 Table Difference in concomitant medication between age≥60 and age <60 in 3 months and 1 year. Abbreviation: ACE, angiotension converting enzyme;
(DOCX)

S2 Table Change of blood pressure at clinic and home during 1 year follow-up by drug dose. Abbreviation: c-, clinic; h-, home; SBP, Systolic blood pressure; DBP, Diastolic blood pressure; *: baseline vs. 3 months, ^†^: baseline vs. 1years, ^‡^: 3 months vs. 1years ^a^ Patients who received the same dose for one year were analyzed.
(DOCX)

S3 Table Decrease in pulse pressure in naïve patients with fimasartan alone through 1 year. (DOCX)

S4 Table Target clinic & home blood pressure achievement rate*^†^ in nonelderly *vs*. elderly. Abbreviation: BP, blood pressure; JNC, Joint National Committee; ESC/ESH, European Society of Cardiology/European Society of Hypertension; ACC/AHA, American College of Cardiology/American Heart Association (AHA).
(DOCX)

S5 Table Difference between pulse pressure reduction for 3 months and 1 year. (DOCX)
