# Supplementary figures and images for: Fimasartan reduces clinic and home pulse pressure in elderly hypertensive patients: A K-MetS study
Source: PLoS One. 2019 Apr 9;14(4):e0214293. doi: 10.1371/journal.pone.0214293 (PMC6456168; doi:10.1371/journal.pone.0214293)

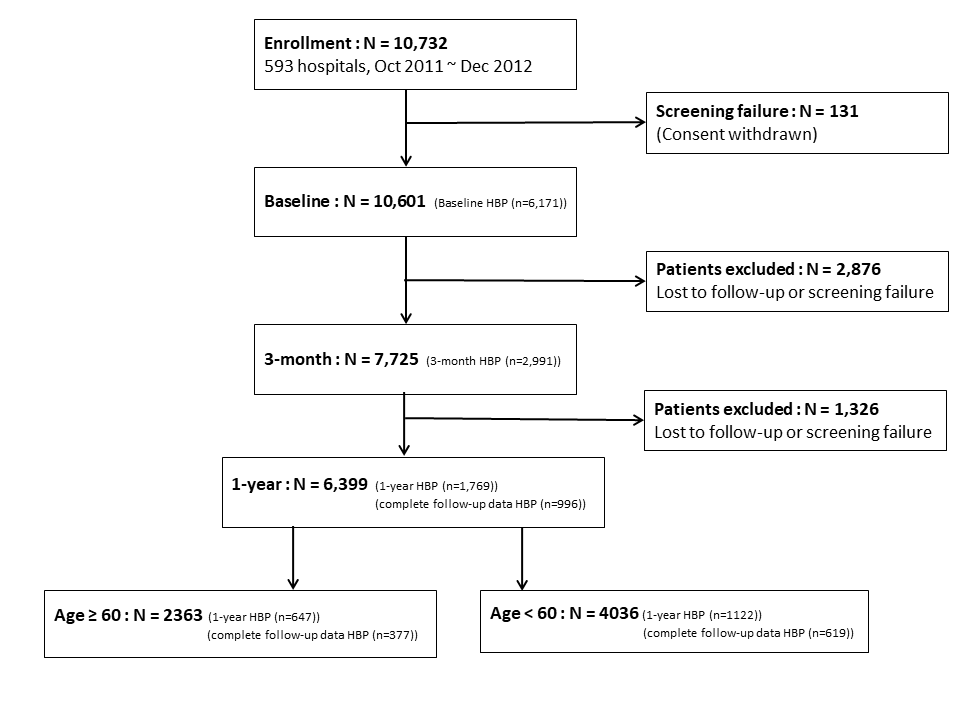

Supplement: S1 Fig — Abbreviation: HBP, high blood pressure. (TIF) [file pone.0214293.s001.tif]

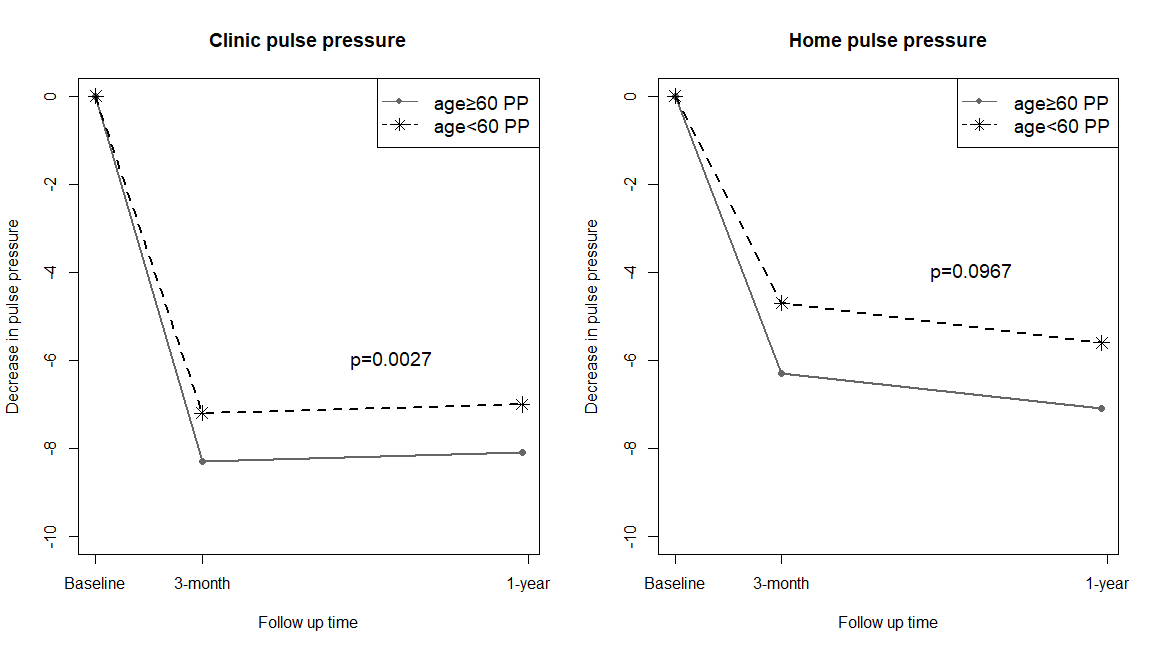

Supplement: S2 Fig — The change in clinic (left panel) and home (right panel) pulse pressure at baseline, 3 months, and 1 year are depicted above. The reduction in clinic pulse pressure between baseline and 3 months and between baseline and 1 year were greater in elderly versus nonelderly patients, when adjusted for sex, body mass index, diabetes mellitus, alcohol, and smoking. P value was obtained by repeated measures analysis of variance. Abbreviation: PP, pulse pressure. (TIF) [file pone.0214293.s002.tif]
