# Supplementary material for: Fimasartan reduces clinic and home pulse pressure in elderly hypertensive patients: A K-MetS study
Source: PLoS One. 2019 Apr 9;14(4):e0214293. doi: 10.1371/journal.pone.0214293 (PMC6456168; doi:10.1371/journal.pone.0214293)
Supplement: S1 Table — Abbreviation: ACE, angiotension converting enzyme; (DOCX) [file pone.0214293.s003.docx]

**S1 Table. Difference in concomitant medication between age**≥**60 and age <60 in 3 months and 1 year.**

|  | Total | Age ≥ 60yr | Age < 60yr | p-value |
| --- | --- | --- | --- | --- |
| **Concomitant medication (%)** |  |  |  |  |
| **3-month** |  |  |  |  |
| ACE inhibitor | 9 (0.1%) | 5 (0.2%) | 4 (0.1%) | 0.4187 |
| Beta blocker | 299 (5%) | 123 (5.5%) | 176 (4.7%) | 0.1443 |
| Calcium channel blocker | 1103 (18.4%) | 469 (21.1%) | 634 (16.8%) | <.0001 |
| Diuretics | 339 (5.6%) | 146 (6.6%) | 193 (5.1%) | 0.0200 |
| Alpha blocker | 19 (0.3%) | 11 (0.5%) | 8 (0.2%) | 0.0986 |
| Other antihypertensive drugs | 48 (0.8%) | 29 (1.3%) | 19 (0.5%) | 0.0013 |
| **1 year** |  |  |  |  |
| ACE inhibitor | 20 (0.3%) | 4 (0.2%) | 16 (0.4%) | 0.1799 |
| Beta blocker | 292 (4.6%) | 127 (5.4%) | 165 (4.1%) | 0.0209 |
| Calcium channel blocker | 1222 (19.2%) | 511 (21.7%) | 711 (17.7%) | 0.0001 |
| Diuretics | 393 (6.2%) | 176 (7.5%) | 217 (5.4%) | 0.0011 |
| Alpha blocker | 19 (0.3%) | 7 (0.3%) | 12 (0.3%) | 1.0000 |
| Other antihypertensive drugs | 42 (0.7%) | 20 (0.8%) | 22 (0.5%) | 0.2017 |

ACE, angiotension converting enzyme;
