# Supplementary material for: Fimasartan reduces clinic and home pulse pressure in elderly hypertensive patients: A K-MetS study
Source: PLoS One. 2019 Apr 9;14(4):e0214293. doi: 10.1371/journal.pone.0214293 (PMC6456168; doi:10.1371/journal.pone.0214293)
Supplement: S2 Table — Abbreviation: c-, clinic; h-, home; SBP, Systolic blood pressure; DBP, Diastolic blood pressure; *: baseline vs. 3 months, †: baseline vs. 1years, ‡: 3 months vs. 1years a Patients who received the same dose for one year were analyzed. (DOCX) [file pone.0214293.s004.docx]

**S2 Table. Change of blood pressure at clinic and home during 1 year follow-up by drug dose.**

|  | Age ≥ 60yr | | | | Age < 60yr | | | | Age≥60yr vs. Age<60yr |
| --- | --- | --- | --- | --- | --- | --- | --- | --- | --- |
|  | Baseline | 3 months | 1 year | p-value | Baseline | 3 months | 1 year | p-value |  |
| **30 mg** | n= 111^a^ | | | | n=183 ^a^ | | | |  |
| c-SBP, mmHg | 138.4 ± 16.6 | 125.8 ± 16.2^*^ | 123.9 ± 13.4^†^ | <.0001 | 138.0 ± 14.6 | 123.3 ± 10.6^*^ | 122.2 ± 11.0^†^ | <.0001 | 0.6936 |
| c-DBP, mmHg | 81.0 ± 10.0 | 75.1 ± 9.7^*^ | 74.2 ± 8.3^†^ | <.0001 | 87.6 ± 9.5 | 78.8 ± 7.1^*^ | 77.2 ± 8.0^†‡^ | <.0001 | 0.0159 |
| h-SBP, mmHg | 140.4 ± 24.6 | 123.7 ± 15.0^*^ | 121.5 ± 9.8^†^ | <.0001 | 137.0 ± 23.7 | 121.7 ± 15.9^*^ | 120.2 ± 10.2^†^ | 0.0001 | 0.0264 |
| h-DBP, mmHg | 79.5 ± 15.5 | 70.6 ± 11.2^*^ | 70.4 ± 7.6^†^ | 0.0007 | 84.1 ± 15.6 | 75.0 ± 11.7^*^ | 74.2 ± 8.1^†^ | <.0001 | 0.2206 |
| **60 mg** | n=1818 ^a^ | | | | n=1044 ^a^ | | | |  |
| c-SBP, mmHg | 143.4 ± 16.5 | 128.0 ± 12.2^*^ | 127.8 ± 12.1^†^ | <.0001 | 143.5 ± 16.5 | 126.9 ± 11.3^*^ | 126.5 ± 11.4^†^ | <.0001 | 0.1237 |
| c-DBP, mmHg | 84.7 ± 10.5 | 77.4 ± 8.4^*^ | 76.6 ± 8.1^†‡^ | <.0001 | 90.0 ± 11.0 | 80.5 ± 8.4^*^ | 79.7 ± 8.2^†‡^ | <.0001 | <0.0001 |
| h-SBP, mmHg | 140.7 ± 19.4 | 128.3 ± 16.2^*^ | 124.8 ± 11.3^†‡^ | <.0001 | 138.9 ± 20.0 | 126.7 ± 17.6^*^ | 123.4 ± 10.1^†‡^ | <.0001 | 0.1215 |
| h-DBP, mmHg | 79.9 ± 12.2 | 73.5 ± 10.4^*^ | 71.7 ± 8.8^†‡^ | <.0001 | 85.3 ± 13.0 | 78.0 ± 11.8^*^ | 76.0 ± 7.7^†‡^ | <.0001 | 0.4805 |
| **120 mg** | n=363 ^a^ | | | | n=212 ^a^ | | | |  |
| c-SBP, mmHg | 147.5 ± 19.6 | 131.0 ± 15.7^*^ | 131.1 ± 14.4^†^ | <.0001 | 148.3 ± 18.7 | 131.0 ± 12.8^*^ | 130.6 ± 11.4^†^ | <.0001 | 0.7639 |
| c-DBP, mmHg | 85.1 ± 11.1 | 76.9 ± 8.6^*^ | 76.6 ± 8.9^†^ | <.0001 | 91.8 ± 11.9 | 81.9 ± 10.3^*^ | 81.2 ± 8.7^†^ | <.0001 | 0.2139 |
| h-SBP, mmHg | 143.9 ± 22.4 | 137.0 ± 22.7^*^ | 134.1 ± 15.1^†^ | 0.0087 | 146.3 ± 23.4 | 136.1 ± 21.6^*^ | 127.1 ± 11.9^†^ | <.0001 | 0.7149 |
| h-DBP, mmHg | 78.7 ± 12.4 | 75.2 ± 10.5^*^ | 72.8 ± 10.0^†^ | 0.0037 | 89.1 ± 14.4 | 83.3 ± 15.5^*^ | 79.0 ± 8.3^†^ | 0.0001 | 0.8361 |

c-, clinic; h-, home; SBP, Systolic blood pressure; DBP, Diastolic blood pressure;

^*^ : baseline vs. 3 months, ^†^ : baseline vs. 1years, ^‡^ : 3 months vs. 1years

^a^ Patients who received the same dose for one year were analyzed.
