# Supplementary material for: Fimasartan reduces clinic and home pulse pressure in elderly hypertensive patients: A K-MetS study
Source: PLoS One. 2019 Apr 9;14(4):e0214293. doi: 10.1371/journal.pone.0214293 (PMC6456168; doi:10.1371/journal.pone.0214293)
Supplement: S3 Table — (DOCX) [file pone.0214293.s005.docx]

**S3 Table. Decrease in pulse pressure in naïve patients with fimasartan alone through 1 year.**

| Drug dose |  | Mean ± SD | | | Adjusted^*^ mean ± SD | | |
| --- | --- | --- | --- | --- | --- | --- | --- |
|  |  | Age ≥ 60yr | Age < 60yr | p-value | Age ≥ 60yr | Age < 60yr | p-value |
| All | Δ(Baseline - 3 months) | n = 415 | n = 871 |  |  |  |  |
|  | Clinic pulse pressure | -11.8 ± 12.5 | -9.8 ± 11.9 | 0.0075 | -11.7 ± 0.6 | -9.9 ± 0.4 | 0.0104 |
|  | Home pulse pressure | -12.5 ± 12.1 | -7.7 ± 8.8 | 0.0003 | -12.2 ± 0.9 | -7.8 ± 0.6 | 0.0001 |
|  | Δ(Baseline - 1 year) |  |  |  |  |  |  |
|  | Clinic pulse pressure | -12.3 ± 12.8 | -9.8 ± 12.4 | 0.0006 | -12.3 ± 0.6 | -9.8 ± 0.4 | 0.0008 |
|  | Home pulse pressure | -12.0 ± 12.7 | -6.9 ± 8.1 | 0.0048 | -11.9 ± 1.3 | -6.9 ± 0.8 | 0.0011 |
|  |  |  |  |  |  |  |  |
| 30 mg | Δ(Baseline - 3 months) | n = 35 | n = 85 |  |  |  |  |
|  | Clinic pulse pressure | -15.0 ± 13.4 | -9.5 ± 9.8 | 0.0330 | -15.0 ± 1.9 | -9.5 ± 1.2 | 0.0155 |
|  | Home pulse pressure | -13.9 ± 12.6 | -8.0 ± 8.1 | 0.0933 | -14.7 ± 2.5 | -7.7 ± 1.5 | 0.0207 |
|  | Δ(Baseline - 1 year) |  |  |  |  |  |  |
|  | Clinic pulse pressure | -13.9 ± 13.6 | -8.4 ± 10.6 | 0.0370 | -13.9 ± 2.0 | -8.4 ± 1.3 | 0.0189 |
|  | Home pulse pressure | -14.1 ± 7.4 | -6.7 ± 6.6 | 0.0081 | -13.2 ± 2.1 | -7.2 ± 1.5 | 0.0342 |
|  |  |  |  |  |  |  |  |
| 60 mg | Δ(Baseline - 3 months) | n = 330 | n = 689 |  |  |  |  |
|  | Clinic pulse pressure | -11.7 ± 12.4 | -10.0 ± 12.2 | 0.0413 | -11.6 ± 0.7 | -10.1 ± 0.5 | 0.0603 |
|  | Home pulse pressure | -11.9 ± 11.5 | -7.5 ± 8.7 | 0.0021 | -11.4 ± 1.0 | -7.7 ± 0.7 | 0.0032 |
|  | Δ(Baseline - 1 year) |  |  |  |  |  |  |
|  | Clinic pulse pressure | -12.2 ± 12.6 | -10.0 ± 12.7 | 0.0110 | -12.1 ± 0.7 | -10.0 ± 0.5 | 0.0132 |
|  | Home pulse pressure | -11.2 ± 13.9 | -6.7 ± 8.1 | 0.0498 | -11.3 ± 1.6 | -6.7 ± 0.9 | 0.0136 |
|  |  |  |  |  |  |  |  |
| 120 mg | Δ(Baseline - 3 months) | n = 50 | n = 97 |  |  |  |  |
|  | Clinic pulse pressure | -10.1 ± 12.3 | -8.7 ± 11.5 | 0.5158 | -9.8 ± 1.7 | -8.8 ± 1.2 | 0.6503 |
|  | Home pulse pressure | -14.1 ± 15.8 | -10.2 ± 13.1 | 0.5437 | -14.0 ± 4.2 | -10.4 ± 5.0 | 0.6107 |
|  | Δ(Baseline - 1 year) |  |  |  |  |  |  |
|  | Clinic pulse pressure | -12.4 ± 13.4 | -9.2 ± 12.2 | 0.1690 | -11.8 ± 1.8 | -9.5 ± 1.3 | 0.2828 |
|  | Home pulse pressure | -14.0 ± 12.6 | -10.1 ± 12.8 | 0.6050 | -10.1 ± 5.7 | -12.5 ± 4.2 | 0.7652 |
