# Supplementary material for: Fimasartan reduces clinic and home pulse pressure in elderly hypertensive patients: A K-MetS study
Source: PLoS One. 2019 Apr 9;14(4):e0214293. doi: 10.1371/journal.pone.0214293 (PMC6456168; doi:10.1371/journal.pone.0214293)
Supplement: S4 Table — Abbreviation: BP, blood pressure; JNC, Joint National Committee; ESC/ESH, European Society of Cardiology/European Society of Hypertension; ACC/AHA, American College of Cardiology/American Heart Association (AHA). (DOCX) [file pone.0214293.s006.docx]

**S4 Table. Target clinic & home blood pressure achievement rate^*†^ in nonelderly *vs*. elderly.**

|  | Total  (n=6399) | Age ≥ 60yr  (n=2363) | Age < 60yr  (n=4036) |
| --- | --- | --- | --- |
| 2014 JNC 8^th^ (150/90 mmHg in age ≥ 60yr) | | | |
| Target BP (n, %) | 5406(84.5) | 2117 (89.6) | 3289 (81.5) |
| Non-target BP (n, %) | 993(15.5) | 246 (10.4) | 747 (18.5) |
| Relative risk = 1.9547, 95% CI = (1.6753 - 2.2800), p-value <.0001 | | | |
|  | Total  (n=6399) | Age ≥ 65yr  (n=1475) | Age < 65yr  (n=4942) |
| 2018 ESC/ESH (140/80 mmHg in age ≥ 65yr) | | | |
| Target BP (n, %) | 3050(47.7) | 778 (53.4) | 2272 (46.0) |
| Non-target BP (n, %) | 3349(52.3) | 679 (46.6) | 2670 (54.0) |
| Relative risk = 1.3464, 95% CI = (1.1976 - 1.5138), p-value <.0001 | | | |
| 2017 ACC/AHA (130/80 mmHg in age ≥ 65yr) | | | |
| Target BP (n, %) | 2561(40) | 590 (40.5) | 1971 (39.9) |
| Non-target BP (n, %) | 3838(60) | 867 (59.5) | 2971 (60.1) |
| Relative risk = 1.0257, 95% CI = (0.9106 - 1.1555), p-value = 0.6745 | | | |

BP, blood pressure; JNC, Joint National Committee; ESC/ESH, European Society of Cardiology/European Society of Hypertension; ACC/AHA, American College of Cardiology/American Heart Association (AHA)
