# Supplementary material for: Fimasartan reduces clinic and home pulse pressure in elderly hypertensive patients: A K-MetS study
Source: PLoS One. 2019 Apr 9;14(4):e0214293. doi: 10.1371/journal.pone.0214293 (PMC6456168; doi:10.1371/journal.pone.0214293)
Supplement: S5 Table — (DOCX) [file pone.0214293.s007.docx]

**S5 Table. Difference between pulse pressure reduction for 3 months and 1 year.**

|  | Baseline - 3 months | Baseline - 1 year | p-value |
| --- | --- | --- | --- |
| Decrease in clinic pulse pressure |  |  |  |
| Age ≥ 60yr | -8.3 ± 13.7 | -8.1 ± 13.8 | 0.4422 |
| Age < 60yr | -7.2 ± 11.9 | -7.1 ± 12.2 | 0.3592 |
| Decrease in home pulse pressure |  |  |  |
| Age ≥ 60yr | -5.9 ± 11.7 | -7.1 ± 12.6 | 0.0091 |
| Age < 60yr | -5.0 ± 9.6 | -5.7 ± 10.5 | 0.0097 |
